# Association of results of the glutaraldehyde coagulation test with plasma acute phase protein concentrations and hematologic findings in hospitalized cows

**DOI:** 10.3389/fvets.2024.1404809

**Published:** 2024-06-19

**Authors:** Florian M. Trefz, Martina Balmer, Laureen M. Peters, Rupert M. Bruckmaier, Mireille Meylan

**Affiliations:** ^1^Clinic for Ruminants, Vetsuisse Faculty, University of Bern, Bern, Switzerland; ^2^Clinic for Ruminants with Ambulatory and Herd Health Services, Centre of Veterinary Clinical Medicine, Ludwig-Maximilians-Universität (LMU) München, Oberschleißheim, Germany; ^3^Clinical Diagnostic Laboratory, Vetsuisse Faculty, University of Bern, Bern, Switzerland; ^4^Veterinary Physiology, Vetsuisse Faculty, University of Bern, Bern, Switzerland

**Keywords:** cows, inflammation, fibrinogen, haptoglobin, serum amyloid A

## Abstract

**Introduction:**

The glutaraldehyde test (GAT) allows for animal-side semi-quantitative estimation of fibrinogen and gamma-globulin concentrations in blood samples of adult cattle and therefore detection of inflammatory disease conditions. However, the test has potential limitations, especially due to the latency period until sufficiently high fibrinogen and/or gamma-globulin concentrations are reached. The aim of the present study was therefore to assess the association between results of GAT with other inflammatory markers including hematologic variables, fibrinogen, plasma haptoglobin and serum amyloid A (SAA) concentrations.

**Methods:**

For the purpose of this prospective observational study, a convenience sample of 202 cows with a broad range of inflammatory and non-inflammatory clinical conditions was included. The GAT was run on EDTA blood, fibrinogen was measured using the Clauss and the heat precipitation method, and commercially available ELISA tests were used for determination of plasma haptoglobin and SAA concentrations.

**Results:**

Shortened GAT coagulation times were more closely correlated to serum globulin (*r_s_* = −0.72) than to plasma fibrinogen concentrations measured with the heat precipitation (*r_s_* = −0.64) and the Clauss method (*r_s_* = −0.70). Cows with a markedly (≤3 min) or moderately (4–6 min) shortened coagulation time had higher (*p* < 0.001) plasma haptoglobin and SAA concentrations than cows with a negative test result. Total leukocyte, monocyte and neutrophil concentrations did not differ significantly between groups. An identified cut-off for the GAT coagulation time of ≤14 min had a sensitivity and specificity of 54.4 and 100%, respectively, for the prediction of an inflammatory state based on clinical findings and/or increased plasma haptoglobin or SAA concentrations.

**Discussion:**

In conclusion, this study demonstrates considerable diagnostic agreement between positive GAT results and increased plasma concentrations of haptoglobin and SAA. Despite high specificity, the test lacks sensitivity in case of acute inflammatory conditions indicating that plasma acute phase protein concentrations and hematologic findings can provide additional diagnostic information if the GAT is negative.

## 1 Introduction

Detection of inflammatory processes is of crucial importance in the clinical management of critically ill animals. In the 1970s, the glutaraldehyde test (GAT) was established as a rapid, cheap, and simple method for animal-side semi-quantitative estimation of fibrinogen and gamma-globulin concentrations in blood samples of adult cattle (1–3). The test is most commonly carried out by mixing equal volumes of a 1.25% glutaric dialdehyde solution with whole blood specimens (usually EDTA blood) in a tube or syringe that is tilted gently in regular intervals until gel formation is observed (4–7). The GAT is based on the chain-linking of protein molecules through their binding of their NH_2_ groups to the aldehyde groups of the reagent, forming polymers which result in macroscopically observable gel formation. The time until gel formation (coagulation time) is directly related to the concentration of fibrinogen and immunoglobulins in the sample, as these protein fractions are characterized by a high content of free amino groups (mainly through lysine residues and aromatic amino acids) (1, 3, 8).

A modification of the test was also used for assessment of passive transfer of colostral immunoglobulins in neonatal calves (9). For this purpose, the test was performed by adding 50 μL of a 10% glutaric aldehyde solution to 0.5 mL of serum. Coagulation times >60 min were indicative of failure of passive transfer (γ-globulin concentrations < 4 g/L) and were also associated with an increased mortality rate during the first month of age (9).

In adult cattle, a coagulation time of the whole-blood GAT of >15 min (based on use of a 1.25% glutaric aldehyde solution in a ratio of 1:1) is generally considered as a negative test result (3, 4, 6, 7), whereas a coagulation time of 7 and 8 min were identified as clinically useful cut-off values to predict an elevation of the total sum of fibrinogen and gamma globulin concentrations of >32 g/L (proportion of correct classifications 82%) (4). Highly shortened coagulation times (≤3 min) are usually associated with serious conditions (mainly chronic bacterial infections or purulent conditions) such as pneumonia, reticuloperitonitis, endo- or pericarditis, pyelonephritis, arthritis, or deep digital sepsis (5, 6, 10–13). In Europe, the GAT has therefore become a popular screening method for detection of inflammatory conditions in the bovine field practice. However, the test has some limitations with regards to the detection of acute inflammatory conditions, as there is a reported latency period of at least 7 days until plasma globulins (which includes both fibrinogen and immunoglobulin concentrations) have increased toward levels that result in a markedly shortened coagulation time (4).

In human and small animal medicine, acute phase proteins (APP) such as C-reactive protein are established as reliable biomarkers of acute inflammatory processes (14, 15). Acute phase proteins are part of the non-specific immune reaction and are produced both by hepatocytes and peripheral tissues after stimulation by cytokines in response to infection, inflammation and tissue damage (15, 16). In this context, haptoglobin and serum amyloid A (SAA) have also attracted attention in bovine medicine in recent years. In cattle, haptoglobin and SAA are considered major APP responders that rapidly increase over 100-fold secondary to an inflammatory stimulus (14–18). These APPs were reported to be effective for measuring the occurrence and severity of the inflammatory response associated with mastitis, enteritis, peritonitis, pneumonia, endocarditis, and endometritis, and elevated levels have also been associated with lameness, ketosis, parturition and the diagnosis of fatty liver syndrome (15, 19–24). However, so far, determination of haptoglobin and SAA have not replaced the GAT in ambulatory rural practice due to the lack of availability of cow-side tests. This led to the question of how far GAT results are reflecting an acute-phase reaction in critically ill cattle. Fibrinogen is an APP in cattle and in other species (15–17, 25), and the GAT should therefore theoretically be altered by an acute phase reaction. However, in contrast to SAA and haptoglobin, fibrinogen is considered a minor to moderate APP responder in cattle, which is only characterized by a 2-fold to maximum 10-fold increase in response to an inflammatory stimulus (15–17, 25). This might therefore result in a quantitatively insufficient increase of total plasma globulin concentrations that are required for a positive GAT result. The most widely used methods for the direct determination of plasma fibrinogen concentrations in cattle (4, 20) include heat precipitation (26) and the coagulometric determination based on the Clauss method (27), but a direct comparison of these methods does not appear to have been carried out in critically ill cattle.

The main objective of the present study was therefore to assess the association between results of whole blood GAT with plasma SAA, haptoglobin, fibrinogen, plasma and serum protein fractions, and hematologic findings in a study population of hospitalized cows with a broad range of clinical conditions. A secondary aim was to perform a method comparison for plasma fibrinogen as determined with the Clauss and heat precipitation methods.

## 2 Materials and methods

### 2.1 Cows

This prospective observational study is based on a convenience sample of 228 cows admitted to the Clinic for Ruminants of the Vetsuisse Faculty of the University of Bern between July 2020 and April 2021. Cows were included in the study regardless of the reason for hospital admission. Specific inclusion criteria were arrival during regular opening times of the Clinical Diagnostic Laboratory and on-clinics periods of the clinicians that were involved in sample collection (MB and FMT). The first 23 enrolled cows were retrospectively excluded due to slight modifications of the study protocol and sampling procedure. Three additional cows were excluded due to an incomplete dataset of laboratory variables. Therefore, a total of 202 cows remained in the study and were included in the analyses reported here. Written informed consent of owners was obtained before study enrollment.

### 2.2 Clinical examination and diagnoses

All cows were managed according to the standard principles of the clinic. After admission to the hospital, cows underwent a thorough clinical examination that included diagnostic imaging procedures (ultrasonographic and/or radiologic examinations) as needed, depending on the presenting complaint and as deemed necessary by the responsible clinician. This problem-based work-up provided the decision basis on whether there was clinical evidence for an inflammatory process or not. If available, intraoperative and/or post-mortem findings were also considered for this purpose. In case of clinical evidence of an inflammation, cows were categorized based on the affected organ system and the primary clinical diagnosis. The latter was determined based on the single most detrimental finding that was considered responsible for the clinical presentation and the condition of the cow. In a next step, clinical inflammatory diagnoses were further subdivided as acute or chronic problems. The identified disorders were considered as acute if at least two of the following criteria were fulfilled:

- History of illness ≤6 days.
- Fever on hospital admission or a history thereof during the last 24 h before hospital admission (rectal temperature ≥ 39.5°C).
- Presence of a neutrophilic left shift during the initial bloodwork.
- Presence of toxic neutrophils observed during the initial bloodwork.

If the criteria for acute inflammation were not met, the identified inflammatory disorders were classified as chronic. Furthermore, an additional definition was used for diagnosis of an inflammatory state based on clinical evidence of an inflammatory process or higher than normal plasma haptoglobin concentrations or higher than normal SAA concentrations (at least one of the three criteria had to be fulfilled). For this purpose, higher than normal plasma haptoglobin concentrations were defined as ≥350 mg/L (19, 28). Normal plasma bovine SAA concentrations were reported to be variable and a reference interval of 0–70 mg/L was recently suggested (18). In the reviewed literature, reported values for healthy dairy cows (control animals or pre-interventional values) using the same analytical assay as in the study reported here were identified as 2.82 ± 1.8 mg/L [*n* = 6; (29)], 4.49 ± 0.57 mg/L [*n* = 20; (20)], 14.24 ± 0.52 mg/L [*n* = 30; (30)], 35.4 ± 6.9 mg/L [*n* = 10; (31)], and 50 ± 52 mg/L [*n* = 20; (32)]. Based on a pooled mean ± SD of 22 ± 25 mg/L from these 86 cows, calculation of the associated 95% confidence interval revealed an upper limit of 71 mg/L. Therefore, a higher than normal plasma SAA concentration was defined as >71 mg/L for the purpose of this study.

### 2.3 Sampling procedures

Blood samples were taken from the jugular vein during initial examination and filled directly in the following order into a plain serum, a citrate, a lithium-heparin, and an EDTA tube (Monovette^®^, Sarstedt, Nürnbrecht, Germany). Samples were submitted within 30 min of collection to the Clinical Diagnostic Laboratory on the campus of the Vetsuisse Faculty Bern for hematologic analysis and determination of fibrinogen concentrations, as well as of serum and plasma total protein, albumin and globulin concentrations.

### 2.4 Laboratory analyses

Total protein concentrations were determined using the Biuret method (TP2, Roche Diagnostics, Basel, Switzerland) and albumin concentrations using the bromcresol green method (ALB2, Roche Diagnostics, Basel, Switzerland) in serum and lithium-heparinized plasma using a clinical chemistry analyzer (Cobas c501, Roche Diagnostics, Basel, Switzerland). Globulin concentrations were calculated by subtraction of albumin concentration from total protein concentration in the corresponding sample. Hematologic variables were determined from an EDTA blood sample using a hematologic analyzer (Advia 2120i, Siemens Healthineers International AG, Zürich, Switzerland). A blood smear was prepared and stained with modified Wright Giemsa on an automated stainer (both Hematek, Siemens Healthineers International AG, Zürich, Switzerland) for a manual 200 cell leukocyte differential count performed by trained laboratory technicians. Fibrinogen concentrations were determined using a coagulometer (STartMax, Stago CH SA, Switzerland) based on the Clauss method (27) and manually with the heat precipitation method (26). For the latter, two microhematocrit tubes were filled with EDTA blood and centrifuged at 16,050 g for 10 min in a microhematocrit centrifuge (Haematokrit 210, Hettich AG, Bäch, Switzerland). Total protein concentration was determined refractometrically in the harvested plasma sample from the first tube, while the second tube was heated for 3 min at 58°C in a heat block. Thereafter, the tube was re-centrifuged for 5 min and the concentration of total protein determined in the supernatant as described. Fibrinogen concentration was calculated by subtracting the total protein concentration of the second tube from that of the first tube. Furthermore, a protein to fibrinogen index was calculated by dividing the total protein concentration from the first tube by the determined plasma fibrinogen concentration.

The GAT was performed by mixing equal volumes of EDTA whole blood and glutaraldehyde solution (1.25% glutaraldehyde and 0.1% Na2-EDTA in 0.9% NaCl) and checking every minute for coagulation by inversion of the test tube. Samples that did not coagulate within a period of minutes of ≤15 min were classified as negative and were assigned a coagulation time of 16 min for statistical analyses as previously described (4).

Commercially available ELISA kits were used for determination of haptoglobin (Bovine Haptoglobin ELISA E-10HPT lot 38, ICL_immunology Consultants Laboratory, Inc., Portland, United States) and SAA concentrations (Multispecies SAA ELISA TP-802, Tridelta Development Ltd., Maynooth, Ireland) on EDTA plasma samples that had been stored at −25°C until analysis. The reported inter- and intra-assay coefficients of variation were 9.9 and 4% for haptoglobin, and 7.8 and 3.9% for SAA, respectively. For analysis of samples, a dilution of 10–10,000 was necessary to obtain reliable results. Both ELISA tests were run at the laboratory of RMB located in Posieux, Switzerland.

### 2.5 Statistical analyses

Statistical analyses were performed using SPSS (version 28.0, IBM, New York, United States) and GraphPad Prism (version 7.01, GraphPad Software, La Jolla, United States). *p*-values ≤0.05 were defined as statistically significant. A normal distribution of data was assessed by the Shapiro–Wilk test and visual inspection of quantile-quantile plots. Because most of the data were not normally distributed, non-parametric tests were used, and data are presented as medians and interquartile ranges (Q_1_ – Q_3_). Spearman’s coefficients of correlation were calculated to determine associations between parameters. Furthermore, linear and power regression models were used to illustrate the association between the GAT coagulation time and plasma or serum protein fractions. For all correlation and regression analyses, only cows with shortened coagulation times ≤15 min were used, as a value of 16 min represented the arbitrarily defined value for a coagulation time greater than 15 min (4). Stepwise forward linear regression models were additionally constructed in order to assess the relative impact of serum globulin and plasma fibrinogen concentrations on the GAT coagulation time. The relative importance of the included variables was assessed by the order of entry into the model as well as by the change in the model R^2^ value (ΔR^2^). Standardized residual plots of each multivariable model were examined to confirm an approximately normal distribution of residuals. Values for the GAT coagulation time were log-transformed to the base 10 because of their mathematical relationship to independent variables and because this approach resulted in a better model fit. Multicollinearity of the final predictors was assessed by calculation of the variance inflation factor; multicollinearity is considered a concern if the variance inflation factor is higher than 5 to 10 (33). Correlation and regression analyses were run with raw and with hematocrit-corrected plasma or serum variables as the GAT coagulation time was based on a whole-blood test. The hematocrit-corrected plasma or serum variables were calculated by use of the following formula (34):


(1) $$\mathrm{Hematocrit}‐\mathrm{corrected}\;\mathrm{variable}=\mathrm{variable}\times \left(1-\mathrm{hematocrit}\right)$$

Cows were grouped according to the GAT coagulation time defined as markedly shortened (≤3 min), moderately shortened (4 to ≤8 min), slightly shortened (9 to ≤15 min), and normal (>15 min). In a previous study (7), coagulation times ≤3 min, >3 to ≤6 min, >6 to ≤15 min, and >15 min were empirically interpreted as indicator of a severe, moderate, mild, and non-detectable inflammatory response. In the present study, a coagulation time of 8 min was however used for differentiation between moderately and slightly shortened test results, because it was identified as a clinically useful cut-off value to detect a higher than normal gammaglobulin and/or fibrinogen concentration (4). Differences between those four groups of cows were assessed using a Kruskal–Wallis test. For the subsequent pair-wise comparisons, the Mann–Whitney *U*-test was used and the level of significance adjusted using the Bonferroni method (total of 6 comparisons, providing an experiment-wise comparison *p* ≤ 0.008). The same approach was also used to compare laboratory findings between groups of cows with clinical evidence of inflammation based on the affected organ system (total of 15 comparisons, providing an experiment-wise comparison significance level at *p* ≤ 0.003).

The diagnostic accuracy of the GAT for prediction of an inflammatory state was assessed by means of a receiver operating characteristic (ROC) analysis. This included the calculation of the area under the ROC curve (AUC-ROC) and the associated 95% confidence interval, as well as the identification of cut-off values that optimized the resulting sensitivity and specificity for the prediction of inflammatory state based on the Youden index (35). The within-day repeatability of GAT was assessed by determining the coefficient of variation (CV) for 20 consecutive measurements each for a purposively selected sample with markedly, moderately, and slightly shortened coagulation times.

Passing-Bablok regression was used to assess the relationship between plasma fibrinogen concentrations as measured with the Clauss and the heat precipitation method. These analyses included the determination of the slope and intercept value of the obtained regression line and associated 95% confidence intervals. Agreement between those two analyses was also examined using Bland–Altman difference plots by displaying the difference in the measured concentrations (*y*-axis) against the mean of those two measurements (*x*-axis). The upper and lower limits of agreement were calculated from the bias ±1.96 x SD (36).

## 3 Results

### 3.1 General conditions and presenting complaints

Most of the cows were Holsteins (*n* = 134, 66%). The remaining breeds were Swiss-Fleckvieh (*n* = 27), Brown-Swiss (*n* = 11), Simmental (*n* = 10), Eringer (*n* = 9), Jersey (*n* = 2), Montbéliard (*n* = 2), Wagyu (*n* = 2), as well as Angus, Galloway, Hinterwälder, Limousin, and a crossbred (*n* = 1 each). Their median age (Q_1_ – Q_3_) was 4.1 (2.9–5.8) years.

The majority of cows (*n* = 122, 60.4%) was referred due to reduced feed intake and/or a suspected gastrointestinal problem which included, among others, 40 cases of abomasal displacement, 22 cases of gastrointestinal ileus, and 17 cases of peritonitis of various origin. The remaining presenting complaints were lameness and orthopedic problems (*n* = 25; 12.3%), udder problems including cases of teat lacerations, teat stenosis, and mastitis (*n* = 25; 12.3%), fever of unknown origin (*n* = 11, 5.4%), respiratory problems (*n* = 8), dystocia (*n* = 5), ophthalmologic problems (*n* = 4), and disorders of the urogenital tract (*n* = 2).

### 3.2 Clinical diagnoses in cows with inflammatory and non-inflammatory disease conditions

A total of 133 cows (65.8%) received a clinical diagnosis of inflammatory disease whereas the clinical work-up revealed no or no clear clinical evidence of an inflammation in the remaining 69 cows. Individual diagnoses of cows with clear clinical evidence of an inflammatory process are listed in Table 1. In these 133 cows, the identified inflammatory conditions were considered as acute in 41 and as chronic in 92 cows. Final diagnoses in the 69 cows with no or no clear clinical evidence of inflammation were gastrointestinal problems (*n* = 42; mainly cases of abomasal displacement and gastrointestinal ileus), teat stenosis (*n* = 8), acute teat or udder injuries (*n* = 4), non-inflammatory musculoskeletal problems (*n* = 4), tumor disease (*n* = 3), dystocia (*n* = 3), ketosis (*n* = 2), as well as epistaxis, nephropathy, and cardiomyopathy (*n* = 1 each).

**Table 1:** Individual diagnoses of 133 cows with clinical evidence of an inflammatory process stratified by organ system.

Individual diagnoses	No. of affected cows
Gastrointestinal inflammatory disease	
Traumatic reticuloperitonitis	24
Peritonitis/intraabdominal adhesions unrelated to reticular foreign bodies	15
Liver abscessation	2
Ruminitis	1
Inflammatory processes in the musculo-skeletal system	
Deep digital sepsis	9
Uncomplicated claw horn lesions	6
Septic arthritis (excl. distal interphalangeal arthritis)	6
Septic bursitis	5
Limb cellulitis	2
Infected limb wounds	2
Infected interdigital hyperplasia	1
Mandibular actinomycosis	1
Inflammatory processes in the urogenital system	
Metritis	20
Endometritis	5
Dystocia (emphysematous calf)	1
Nephritis	1
Udder inflammatory disease	
Clinical mastitis	14
Respiratory inflammatory disease	
Pneumonia	7
Miscellaneous inflammatory processes	
Intertrigo	2
Retropharyngeal abscessation	3
Periphlebitis	2
Mediastinal abscessation	1
Abdominal wall cellulitis	1
Tooth root abscess	1
Conjunctivitis	1

### 3.3 Results of the glutaraldehyde test and its association with serum and plasma protein fractions, APP, and hematologic findings

The GAT coagulation time was normal in 82 cows (40.6%), slightly shortened in 49 cows (24.3%), moderately shortened in 45 cows (22.3%), and markedly shortened in 26 cows (12.9%).

Median and interquartile ranges for plasma and serum protein fractions, APP concentrations, and hematologic variables in those four groups of cows are reported in Table 2. Cows with markedly or moderately shortened GAT coagulation times had significantly higher plasma and serum total protein and globulin concentrations as well as significantly higher plasma fibrinogen, haptoglobin, and SAA concentrations than cows with slightly reduced or normal GAT coagulation times. In contrast, cows with a markedly or moderately shortened coagulation time had significantly lower plasma and serum albumin concentrations resulting in a significantly lower albumin to globulin ratio than in cows with a slightly shortened or normal GAT coagulation time. Leukocyte, monocyte and neutrophil counts did not differ significantly among groups (Table 2).

**Table 2:** Median and interquartile ranges for plasma and serum protein fractions, plasma acute phase protein concentrations, and hematologic variables in 202 hospitalized cows categorized by results of the coagulation time of a whole-blood glutaraldehyde test.

		Glutaraldehyde test coagulation time				
Variable	Reference interval	Markedly shortened (≤3 min) *n* = 26	Moderately shortened (4–8 min) *n* = 45	Slightly shortened (9–15 min) *n* = 49	Normal (>15 min) *n* = 82	*p*-value
Plasma proteins						
Total protein (g/L)	[64–82]	92.0 (87.8/100.3)^a^	83.0 (78.7/88.9)^b^	77.2 (72.8/83.1)^c^	75.0 (70.5/79.4)^c^	<0.001
Albumin (g/L)	[30–41]	27.5 (26.3/31.0)^a^	31.1 (27.5/33.6)^b^	34.7 (31.8/37.6)^c^	36.3 (33.2/37.7)^c^	<0.001
Globulin (g/L)	[28–51]	61.7 (60.2/74.6)^a^	52.1 (49.1/57.8)^b^	43.6 (40.3/47.9)^c^	39.4 (35.0/44.2)^d^	<0.001
Albumin to globulin ratio	[n.a.]	0.44 (0.35/0.51)^a^	0.60 (0.50/0.65)^b^	0.77 (0.71/0.86)^c^	0.91 (0.80/1.03)^d^	<0.001
Serum proteins						
Total protein (g/L)	[60–80]	82.8 (75.7/91.0)^a^	74.5 (72.2/80.9)^a^	69.7 (66.0/77.1)^b^	68.9 (65.5/74.0)^b^	<0.001
Albumin (g/L)	[30–40]	30.8 (28.1/32.8)^a^	33.2 (29.9/34.9)^b^	35.5 (33.3/38.8)^c^	37.0 (34.4/39.0)^c^	<0.001
Globulin (g/L)	[30–40]	51.2 (48.1/61.8)^a^	43.2 (39.5/47.8)^b^	35.4 (32.2/39.5)^c^	33.4 (28.8/37.5)^d^	<0.001
Albumin to globulin ratio	[n.a.]	0.60 (0.48/0.67)^a^	0.75 (0.65/0.83)^b^	0.99 (0.87/1.11)^c^	1.12 (1.01/1.31)^d^	<0.001
Plasma acute phase proteins						
Fibrinogen Clauss (g/L)	[1–6]	7.6 (6.8/8.4)^a^	5.9 (5.0/7.4)^b^	4.9 (3.8/5.5)^c^	3.8 (3.2/4.7)^d^	<0.001
Fibrinogen Heat (g/L)	[1–6]	10 (8/10)^a^	7 (6/8)^b^	6 (4/6)^c^	4 (4-6)^c^	<0.001
Protein to fibrinogen index	[>11]	9.6 (8.3/11.7)^a^	12.1 (9.8/15.0)^a,b^	14.3 (11.9/18.8)^b,c^	16.9 (12.7/21.5)^c^	<0.001
Haptoglobin (mg/L)	[<350]	3,288 (1,314/5,965)^a^	1,297 (394/3,060)^a^	400 (2/2,155)^b^	40 (0.8/612)^b^	<0.001
Serum amyloid A (mg/L)	[≤71]	650 (279/1094)^a^	530 (188/907)^a^	183 (59/480)^b^	156 (23/439)^b^	<0.001
Complete blood count						
Leukocytes (×10^9^/L)	[4–10]	7.3 (5.7/10.0)^a^	7.4 (5.6/10.6)^a^	9.6 (7.1/11.6)^a^	7.5 (5.7/10.9)^a^	0.096
Segmented neutrophils (×10^9^/L)	[1.0–3.5]	4.1 (3.1/7.1)^a^	4.6 (2.9/6.6)^a^	5.3 (3.5/7.1)^a^	4.1 (2.9/6.0)^a^	0.21
Band neutrophils (×10^9^/L)	[0.0–0.2]	0.0 (0.0/0.05)^a^	0.0 (0.0/0.12)^a^	0.0 (0.0/0.13)^a^	0.0 (0.0/0.06)^a^	0.57
Monocytes (×10^9^/L)	[0.0–0.33]	0.30 (0.14/0.47)^a^	0.35 (0.18/0.54)^a^	0.29 (0.16/0.56)^a^	0.30 (0.19/0.51)^a^	0.96
Lymphocytes (×10^9^/L)	[2.5–5.5]	2.3 (2.0/3.5)^a^	2.4 (1.8/3.1)^a^	2.9 (2.4/3.6)^a^	2.6 (2.0/3.1)^a^	0.075

Reported *p*-values are based on a Kruskal–Wallis test, which was used to assess differences between the four groups of cows. Different letters indicate a statistically significant difference between groups based on pair-wise comparisons using a Mann–Whitney *U*-test (*p* ≤ 0.008). Reference intervals for serum biochemistry according to (37). See section 2.2 for normal values regarding serum amyloid A and haptoglobin concentrations. The remaining reference values are based on normal values of the Clinical Diagnostic Laboratory of the Vetsuisse Faculty Bern. Fibrinogen Clauss = plasma fibrinogen concentration determined with the Clauss method, Fibrinogen Heat = plasma fibrinogen concentration determined with the heat precipitation method, n.a., not available.

The GAT coagulation time was shortened (≤15 min) in 82/106 cows (77.4%) with increased plasma haptoglobin and 103/155 cows (66.5%) with increased plasma SAA concentrations. At least one hematologic abnormality with regards to the presence of monocytosis, neutrophilia, neutropenia, left shift or toxic changes of neutrophils was seen in 161/202 cows (79.7%). The GAT coagulation time was shortened in 57/92 cows (62.0%) with monocytosis, 78/129 cows (60.5%) with neutrophilia, 1/3 cows (33.3%) with neutropenia, 20/33 cows (60.6%) with a neutrophil left shift, and 8/13 cows (61.5%) with toxic neutrophils.

**Figure 1:**
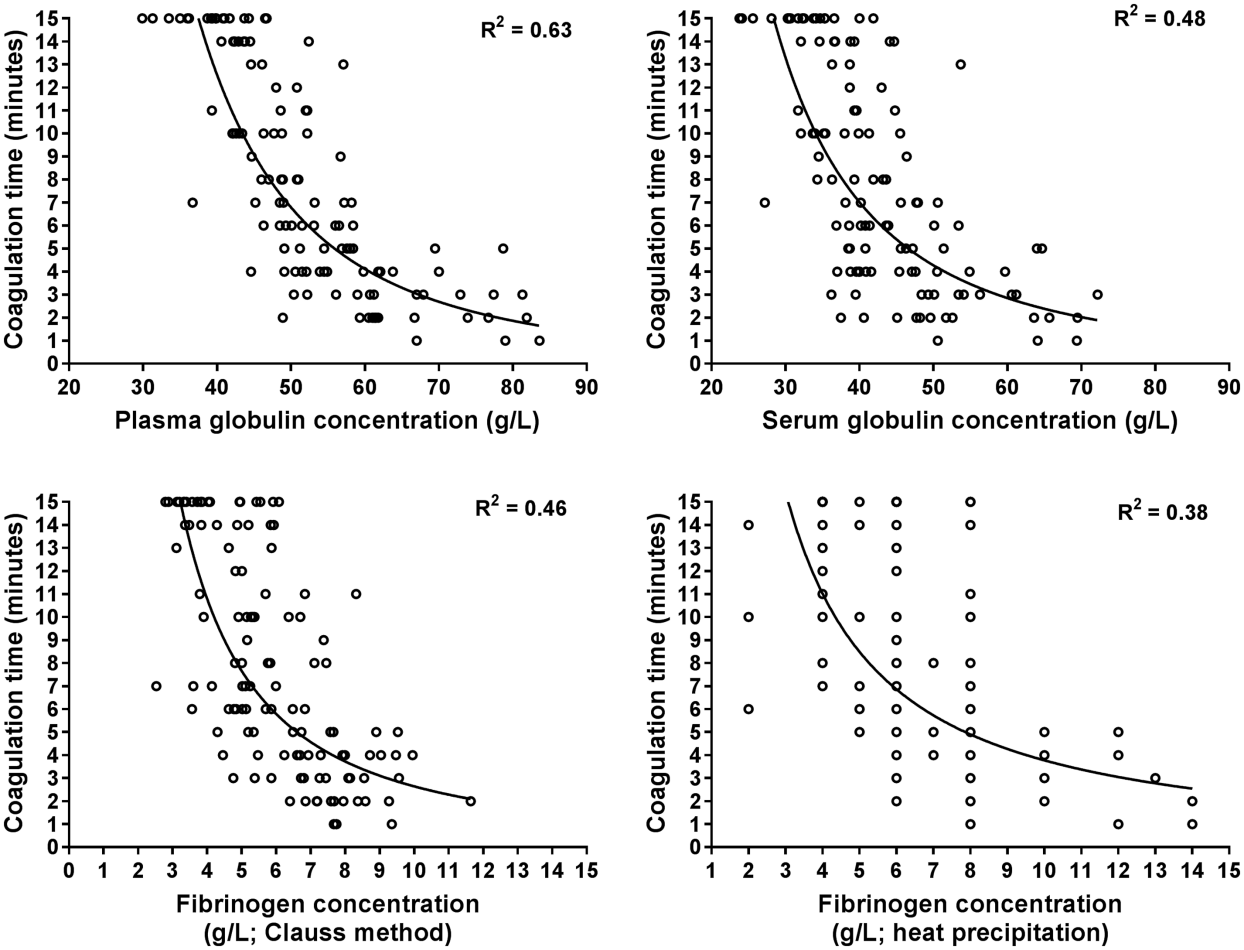
Scatterplots illustrating the relationship between the coagulation time of the whole blood glutaraldehyde test with plasma and serum globulin concentrations as well as with plasma fibrinogen concentrations as determined with the Clauss and heat precipitation methods. The plots are based on 120 data points from hospitalized cows with a shortened coagulation time (≤15 min). Many data points of fibrinogen concentration determined with the heat precipitation method are superimposed as it was measured in steps of 1 g/L. The lines represent the results of power regression analysis.

**Figure 2:**
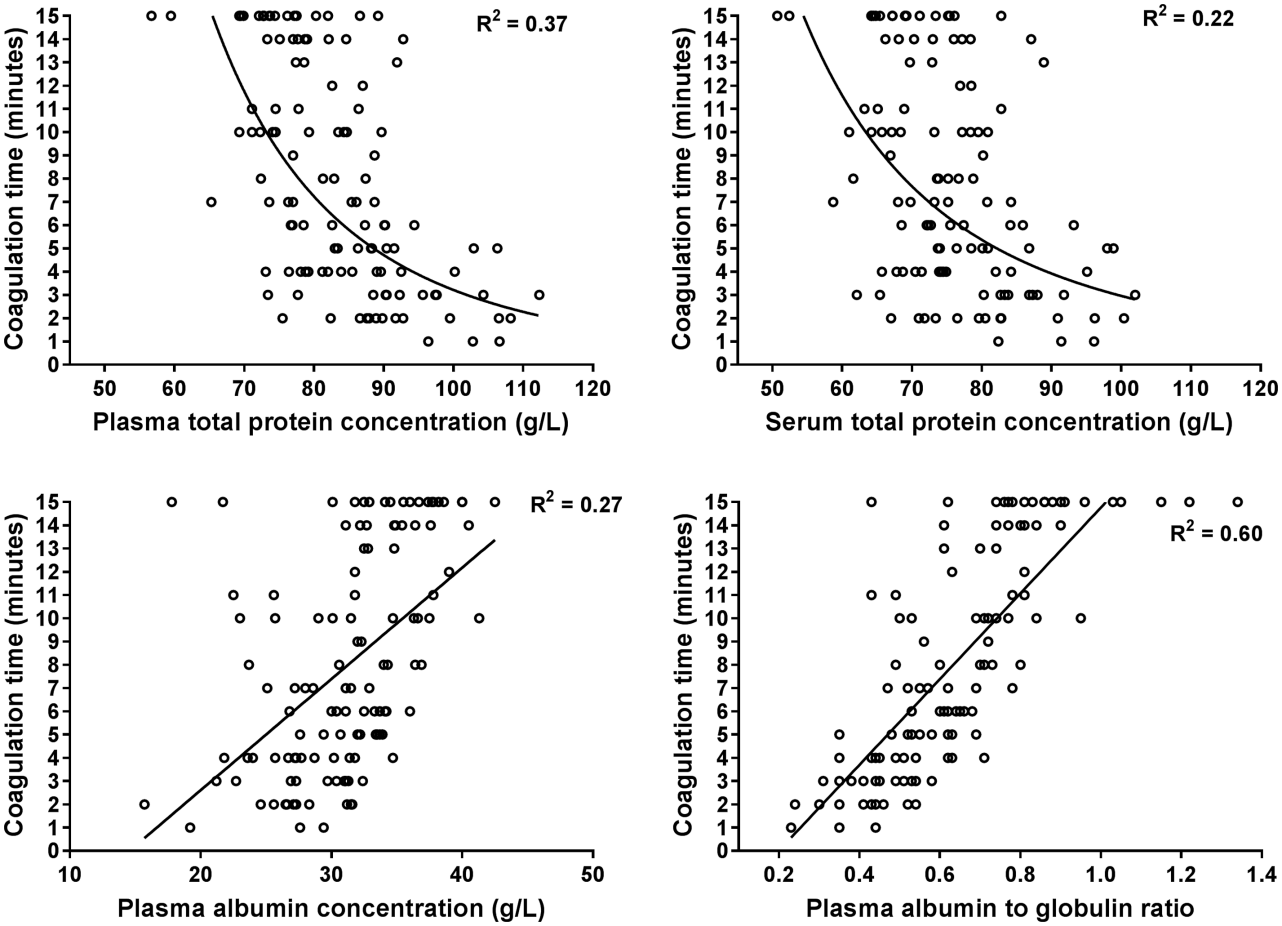
Scatterplots illustrating the relationship between the coagulation time of the whole blood glutaraldehyde test with plasma and serum total protein concentrations, plasma albumin concentrations, and the plasma albumin to globulin ratio. The plots are based on 120 data points from hospitalized cows with a shortened coagulation time (≤15 min). The lines represent the results of power (plasma and serum total protein) and linear regression analysis (plasma albumin and albumin to globulin ratio).

The GAT coagulation time was negatively correlated to total protein and globulin concentrations, as well as fibrinogen, haptoglobin and SAA concentrations (Table 3). Selected scatterplots are shown in Figures 1, 2 and respective regression functions are reported in Supplementary Table S1. A shortened GAT coagulation time was most closely correlated to plasma globulin concentrations and plasma albumin to globulin ratios. The power regression function for plasma globulin concentrations was:


(2) $$\mathrm{GAT}=321998\;\times \;{\left(\mathrm{plasma}\;\mathrm{globulin}\;\mathrm{concentration}\right)}^{-2.752}$$

**Table 3:** Spearman’s coefficients of correlation between plasma and serum protein fractions, plasma acute phase protein concentrations, and the coagulation time of a whole-blood glutaraldehyde test (GAT) in 120 hospitalized cows with a shortened coagulation time (≤15 min).

	GAT coagulation time	
Variable	r_s_^*^ Raw values	r_s_^*^ Hct-corrected values
Plasma proteins		
Total protein	−0.61	−0.74
Albumin	0.58	0.45
Globulin	−0.83	−0.89
Albumin to globulin ratio	0.80	0.74
Serum proteins		
Total protein	−0.46	−0.62
Albumin	0.52	0.39
Globulin	−0.72	−0.80
Albumin to globulin ratio	0.76	0.69
Plasma acute phase proteins		
Fibrinogen Clauss	−0.70	−0.75
Fibrinogen Heat	−0.64	−0.70
Haptoglobin	−0.54	−0.55
Serum amyloid A	−0.46	−0.48

^*^P-values were <0.001 for all reported coefficients of correlation. Coefficients were calculated with raw and hematocrit-corrected concentrations of laboratory variables (see equation 1).

A numerically higher coefficient of correlation was found for the association between GAT coagulation times and serum globulin concentrations when compared to obtained coefficients for the association between GAT coagulation times and fibrinogen concentrations. Stepwise linear regression analysis indicated that the serum globulin concentrations provided higher explanatory power for the prediction of shortened GAT coagulation times than plasma fibrinogen concentrations as determined with either the Clauss or the heat precipitation methods (Table 4).

**Table 4:** Results of a stepwise linear regression analysis for the prediction of a shortened coagulation time of the glutaraldehyde test in 120 hospitalized cows by means of hematocrit-corrected concentrations for serum globulins and plasma fibrinogen concentrations as determined with the heat precipitation (fibrinogen heat) and Clauss method (fibrinogen Clauss).

Order of entry	Variable^*^	∆*R*^2^	Model *R*^2^	Coefficient	±SE	*p*-value	Variance inflation factor
Model based on fibrinogen Clauss							
	Constant	–	–	1.992	0.063	<0.001	–
1	Serum globulin	0.568	0.568	−0.023	0.002	<0.001	1.23
2	Fibrinogen	0.201	0.769	−0.115	0.011	<0.001	1.23
Model based on fibrinogen Heat							
	Constant	–	–	1.976	0.06	<0.001	–
1	Serum globulin	0.568	0.568	−0.025	0.002	<0.001	1.15
2	Fibrinogen	0.218	0.786	−0.087	0.008	<0.001	1.15

^*^Serum globulin and plasma fibrinogen concentrations were corrected for hematocrit by multiplying values with a factor based on 1-hematocrit. Values for the GAT coagulation time were log-transformed to the base 10 for analyses. ∆*R*^2^ indicates the increase in the model *R*^2^ caused by the addition of the variable to the multivariable linear regression model.

### 3.4 Results of the whole-blood glutaraldehyde test, plasma APP concentrations and hematologic findings in cows diagnosed with an inflammatory disease condition

**Figure 3:**
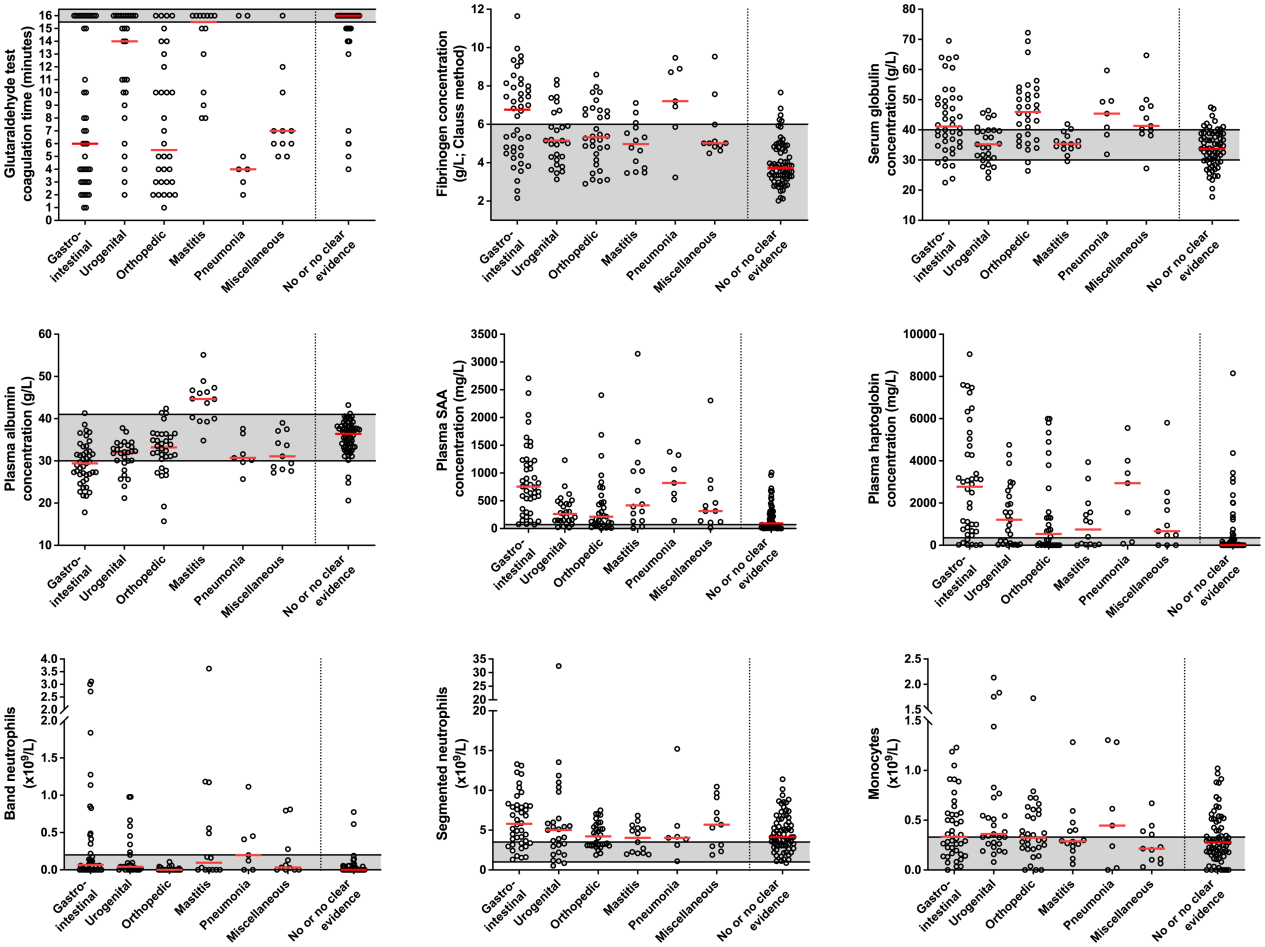
Coagulation times of the glutaraldehyde test, plasma acute phase protein concentrations and results of the leukocyte differential counts in cows diagnosed with an inflammatory gastrointestinal (*n* = 42), orthopedic (*n* = 32), urogenital (*n* = 27), udder (*n* = 14), respiratory (*n* = 7), and miscellaneous problem (*n* = 11) and in those cows which had no or no clear clinical evidence of inflammation (*n* = 69). Median values in each group are indicated by solid red lines. Gray areas represent the reference interval of the respective variable. See Table 1 for individual diagnoses of cows that were diagnosed with an inflammatory process.

Coagulation times of GAT, plasma APP concentrations and results of the leukocyte differential counts in cows diagnosed with an inflammatory gastrointestinal, orthopedic, urogenital, udder, respiratory, or miscellaneous problems and in those cows which had no or no clear evidence of inflammation are illustrated in Figure 3.

A statistically significant difference among disease groups was found for GAT coagulation times (*p* < 0.001), plasma albumin concentrations (*p* = 0.013), fibrinogen as measured with heat precipitation (*p* = 0.005), haptoglobin (*p* = 0.01), SAA (*p* < 0.001), and band neutrophil concentrations (*p* < 0.001).

Cows diagnosed with an inflammatory gastrointestinal condition had significantly lower GAT coagulation times (*p* = 0.001) and plasma albumin concentrations (*p* = 0.002) when compared to cows with clinical mastitis, but higher plasma SAA concentrations when compared to cows with urogenital disorders (*p* < 0.001), as well as higher plasma haptoglobin (*p* = 0.001) and SAA concentrations (*p* < 0.001) when compared to cows with an inflammatory orthopedic problem. Plasma albumin concentrations also differed between cows with an inflammatory gastrointestinal and orthopedic problem (*p* = 0.002). Cows with an inflammatory orthopedic problem had significantly lower GAT coagulation times when compared to cows with clinical mastitis (*p* < 0.001), and significantly lower band neutrophil counts when compared to cows with inflammatory gastrointestinal (*p* < 0.001), respiratory (*p* = 0.003), and urogenital disorders (*p* < 0.001), as well as mastitis (*p* = 0.002). Cows that were diagnosed with pneumonia had significantly higher plasma fibrinogen concentrations as measured with heat precipitation (*p* = 0.002) than cows with urogenital and orthopedic problems.

A comparison of laboratory findings between all cows diagnosed with an acute and those with a chronic inflammatory condition can be found in Table 5. Cows that were considered to be in an acute inflammatory state had significantly lower plasma and serum total protein and globulin concentrations, but significantly higher GAT coagulation times, leukocyte concentrations, and plasma SAA concentrations when compared to cows with a chronic condition. Plasma fibrinogen and haptoglobin concentrations did not differ between acute and chronic disease groups. Spearman’s coefficients of correlation between plasma SAA and haptoglobin were 0.45 (*p* = 0.003) and 0.75 (*p* < 0.001) for cows with acute and chronic inflammatory disease conditions, respectively.

**Table 5:** Comparison of laboratory findings between cows that were diagnosed with an acute and those with a chronic inflammatory condition.

Variable	Cows with acute inflammatory conditions (*n* = 41)	Cows with chronic inflammatory conditions (*n* = 92)	*p*-value
Glutaraldehyde test (min)	16 (10/16)	6 (3/12)	<0.001
Plasma proteins			
Total protein (g/L)	75.2 (70.8/78.0)	85.1 (77.9/90.2)	<0.001
Albumin (g/L)	31.8 (28.5/36.4)	31.5 (27.3/34.5)	0.53
Globulin (g/L)	42.4 (37.8/47.6)	52.2 (46.8/60.9)	<0.001
Albumin to globulin ratio	0.74 (0.62/0.93)	0.59 (0.46/0.73)	<0.001
Serum proteins			
Total protein (g/L)	67.0 (62.9/72.0)	76.8 (71.5/82.8)	<0.001
Albumin (g/L)	33.6 (31.5/37.4)	33.5 (29.8/35.5)	0.28
Globulin (g/L)	33.3 (28.8/38.1)	43.4 (37.9/50.1)	<0.001
Albumin to globulin ratio	1.06 (0.78/1.21)	0.74 (0.63/0.92)	<0.001
Plasma acute phase proteins			
Fibrinogen Clauss (g/L)	5.3 (4.3/6.3)	5.4 (4.5/7.4)	0.16
Fibrinogen Heat (g/L)	6 (5/8)	6 (5/8)	0.50
Protein to fibrinogen index	12.3 (9.3/14.7)	12.9 (9.6/15.4)	0.25
Haptoglobin (mg/L)	1,553 (338/3,149)	977 (27/3,104)	0.24
Serum Amyloid A (mg/L)	550 (289/1,144)	310 (123/812)	0.01
Complete blood count			
Leukocytes (×10^9^/L)	9.2 (6.4/12.9)	8.1 (5.7/10.0)	0.038
Segmented neutrophils (×10^9^/L)	5.0 (3.2/8.1)	4.5 (3.0/6.5)	0.26
Band neutrophils (×10^9^/L)	0.45 (0.03/0.91)	0.0 (0.0/0.06)	<0.001
Monocytes (×10^9^/L)	0.32 (0.25/0.65)	0.34 (0.19/0.55)	0.30
Lymphocytes (×10^9^/L)	2.5 (1.9/3.4)	2.5 (1.9/3.2)	0.83

See Table 2 for reference values. Values are reported as medians and interquartile ranges. *P*-values are based on a Mann–Whitney *U*-test.

### 3.5 Accuracy of the whole blood glutaraldehyde coagulation test to predict an inflammatory state based on clinical findings and/or higher than normal plasma haptoglobin or SAA concentrations

An inflammatory state based on clinical findings and/or laboratory evidence based on higher than normal plasma SAA or haptoglobin concentrations was diagnosed in 169 cows (83.7%). From these 169 cows, 89 had clinical evidence of inflammation plus higher than normal plasma SAA and haptoglobin concentrations, 30 had clinical evidence plus higher than normal plasma SAA concentrations, and one cow had clinical evidence of an inflammatory process plus higher than normal plasma haptoglobin concentration. Thirteen additional cows had only clinical evidence of inflammation, and 36 cows had only laboratory evidence based on increased SAA (*n* = 20) or increased plasma SAA and haptoglobin concentrations (*n* = 16). The AUC-ROC curve for GAT was 0.76 (95% CI, 0.69–0.83; *p* < 0.001) and an identified cut-off for the coagulation time of ≤14 min had a sensitivity and specificity of 54.4 and 100%, respectively, for the prediction of an inflammatory state based on these criteria.

### 3.6 Method comparison for measurement of plasma fibrinogen concentrations

**Figure 4:**
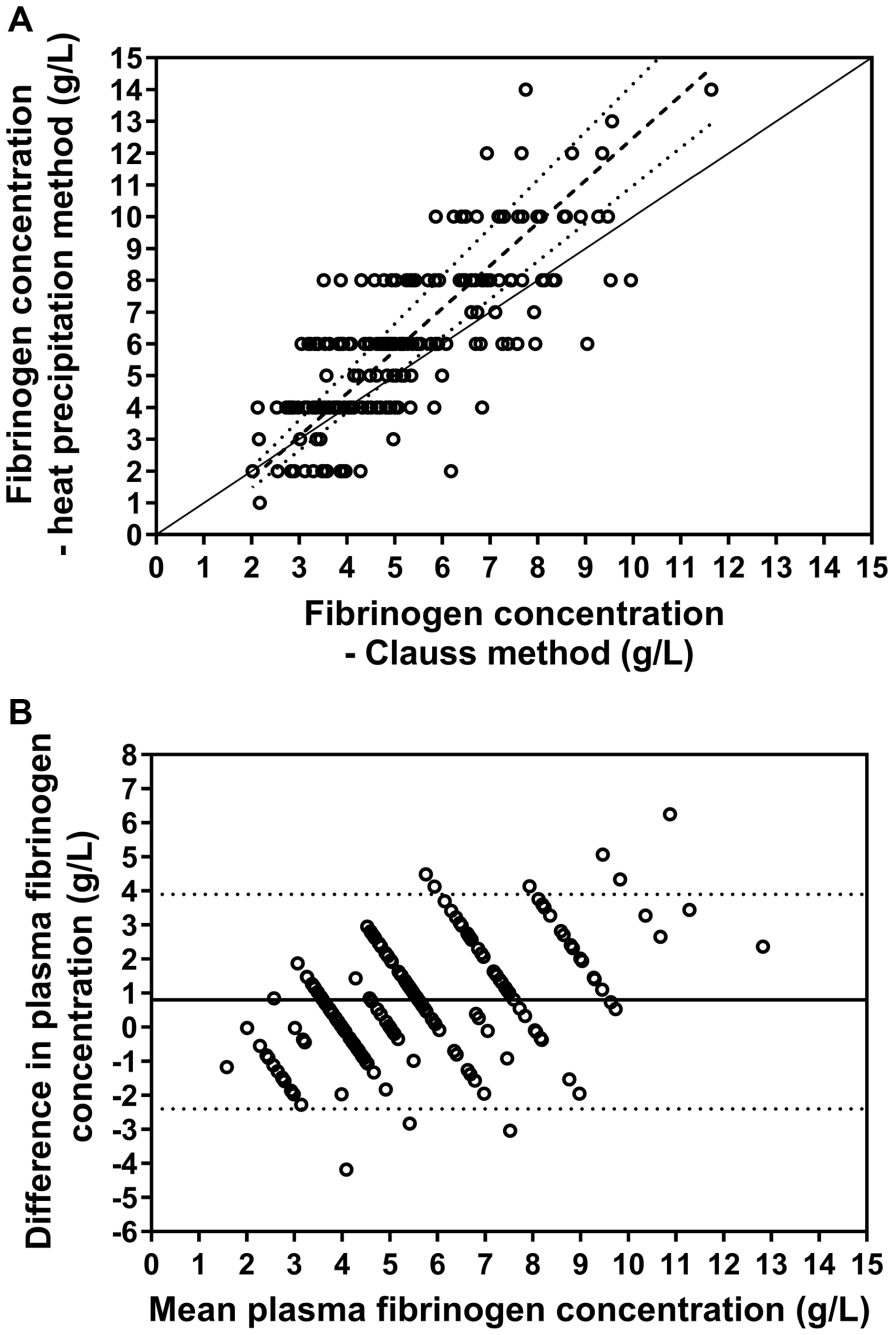
**(A)** Relationship between plasma fibrinogen concentration measured manually with the heat precipitation method and determined using the Clauss method (*n* = 202). The solid line represents the line of identity, the dashed line the results of Passing-Bablok regression and the dotted lines the resulting 95% confidence intervals of the slope. **(B)** Bland–Altman plot displaying the agreement of those two measurements. The solid horizontal line represents the mean bias and the dotted lines the 95% confidence limit of agreement. The analysis revealed that the heat precipitation method measured plasma fibrinogen on average 0.8 g/L higher than the Clauss method.

Passing-Bablok regression analysis for the agreement between plasma fibrinogen concentrations measured by the heat precipitation method and Clauss method indicated a proportional bias of −0.92 (95% CI: −1.73 to −0.12) and a constant bias of 1.34 (95% CI: 1.19–1.51) (Figure 4A). The Bland–Altman difference plot (Figure 4B) indicated a mean bias of 0.8 g/L, i.e., that the heat precipitation method measured plasma fibrinogen on average 0.8 g/L higher than the Clauss method. The 95% limits of agreement were −2.4 to 3.9 g/L. The resulting bias was positively associated (*r_s_* = 0.41, *p* < 0.001) with respective mean values of fibrinogen as measured by those two methods.

### 3.7 Repeatability of the glutaraldehyde test

The within-day CV of the GAT in three whole blood samples with a coagulation time of 10, 6, and 3 min were 10.8, 11.5, and 10.8%, respectively.

## 4 Discussion

Central findings of this study indicate that there is considerable diagnostic agreement between positive GAT results and increased plasma concentrations of haptoglobin and SAA, therefore confirming findings of previous studies that the GAT represents a clinically useful screening test for cow-side detection of inflammatory disease conditions (4–7). Results of the performed regression analyses indicate that shortened GAT coagulation times were most closely associated with higher than normal plasma globulin concentrations and a lower than normal plasma or serum albumin to globulin ratio, which is also in line to findings of previous studies in cattle (3, 4, 6, 7, 34) and horses (38). Use of the obtained power regression function for plasma globulin concentration (Equation 2) indicates that a coagulation time of 6 min predicts an abnormal plasma globulin concentration of ≥52 g/L (Figure 1), which is similar to a reported cut-off value of 7 min in a previous study to predict a higher than normal total sum of gamma-globulin and fibrinogen concentrations (4).

The plasma albumin to globulin ratio is generally considered to represent an overall proxy of abnormal protein status in dairy cattle (39) which was reliably reflected by GAT coagulation times. In the context of cow-side detection of abnormal protein status the GAT was also previously called “pocket electrophoresis” (4). Plasma globulins consist of the α-fraction which includes haptoglobin, the ß-fraction which includes fibrinogen, and the γ-fraction which is represented by most immunoglobulins (39, 40). Albumin is known as a negative APP characterized by decreasing concentrations during an acute phase reaction as a result of decreased hepatic albumin synthesis or extravasation due to endotoxemia (16, 25, 41). Leakage of albumin into the abdominal cavity (42) was additionally considered as a likely frequent reason for hypoalbuminemia in cows of this study, because peritonitis was the most commonly observed diagnosis and lowest values for albumin concentrations were seen in cows with an inflammatory gastrointestinal disorder (Figure 3).

A clinically relevant question is whether the GAT allows to detect acute inflammatory conditions causing a rise of plasma fibrinogen as the result of an acute phase reaction before an increase in immunoglobulin concentrations. Previous studies have shown that increased fibrinogen concentrations contribute to a shortening of GAT coagulation times (4, 34, 43). The finding of a higher coefficient of correlation between a shortened GAT coagulation and plasma globulin when compared to serum globulin concentration in the present study supports this conclusion. Furthermore, plasma fibrinogen concentrations provided additional explanatory power to serum globulin concentrations for the prediction of a shortened GAT coagulation time in the performed multivariable linear regression analyses (Table 4). Nevertheless, fibrinogen is only a minor to moderate acute phase responder (15–17, 25) resulting in a numerically lower increase of fibrinogen levels when compared to the potential rise of serum globulin concentrations as seen with chronic inflammatory conditions in the present study (Tables 2, 5). However, fibrinogen is characterized by a more than twice as high molecular mass and a higher content of lysine and aromatic acid residues when compared to gamma-globulins (8, 40), potentially facilitating crosslinking of molecules with glutaraldehyde (34). By comparing GAT coagulation time between whole blood and serum specimens, the results of a previous study gave evidence that fibrinogen is indeed characterized by a faster reaction kinetic to the glutaraldehyde test solution when compared to immunoglobulins (34). This assumption was also based on the observation of fivefold shorter GAT coagulation times when the test was run on plasma rather than on serum, as both specimens do not contain erythrocytes and differ only in their fibrinogen content (4, 34). For those reasons, it was proposed that acute inflammatory conditions should also result in a shortening of the whole blood GAT (34). This assumption was however not supported by findings of the present study, where a marked and statistically significant difference in terms of GAT coagulation times between acute (median: 16 min) and chronic inflammatory conditions (median: 6 min) was observed, despite similar plasma fibrinogen concentrations in those groups of cows (Table 5). This is also in line with a 1978 study (43), which demonstrated that, in the presence of normal immunoglobulin concentrations, the GAT was only positive in a group of cows with markedly increased plasma fibrinogen concentration (9.6 ± 2.7 g/L), whereas a moderate rise of fibrinogen (5.7 ± 1.6 g/L), as compared to a healthy control group (4.0 ± 0.7 g/L), did not result in a positive whole blood GAT reaction.

Our comparison analysis for fibrinogen measurement methods revealed that the heat precipitation method measured on average 0.8 g/L higher fibrinogen concentrations than the coagulometric determination with the Clauss method. Furthermore, Passing-Bablok regression indicated a proportional bias between those two measurements, in other words, the observed bias increased with increasing fibrinogen concentrations. Similar findings were also reported in a study in horses (44). Although both methods claim to measure the same, the underlying principles leading to the obtained results are, however, very different and in our opinion not fully comparable. The heat precipitation is based on the refractive index of total solids in a solution (including mainly protein, but also other dissolved solids such as glucose and urea), which is converted to total protein concentration by subtracting a fixed concentration of expected non-protein solids. Therefore, if concentrations of non-protein solids significantly exceeds physiological levels, they can falsely increase the total protein measurement (40). Additionally, although fibrinogen is the major component of solids that precipitate after heating to 56°C, other solids may precipitate as well, so we cannot be certain that we measured fibrinogen specifically. Furthermore, reading the scale on a refractometer can be inaccurate (e.g., in the presence of lipemia) and operator dependent, thus the heat precipitated fibrinogen measurement has a gray zone of approximately +/− 2 g/L (40). The Clauss method, on the other hand, measures the functional activity of fibrinogen and converts this to fibrinogen concentration based on a standard curve (27), for which a human plasma calibrator was used, which may not be directly applicable to other species. Use of a species-specific standard curve may have been more appropriate, but this is not offered in our laboratory. Furthermore, interference with coagulability by fibrinogen degradation products was reported in dogs, which was responsible for underestimation of increased fibrinogen concentrations measured with the Clauss method (45). Additionally, dysfibrinogenemia, as seen with some hepatopathies, can lead to dysfunctional fibrin, in which cases coagulability does not correlate with concentration (46, 47). But taken together, the results of the Clauss method was considered more accurate than the heat precipitation in the present study. This assumption is supported by the finding that fibrinogen concentration as measured with the Clauss method was more closely correlated to shortened GAT coagulation time than it was the case for fibrinogen concentrations as measured with the heat precipitation method (Table 3).

A lack of sensitivity of the GAT was confirmed in the present study when test results were used to predict an inflammatory disease state based on clinical findings and/or higher than normal plasma SAA or haptoglobin concentrations. This is underscored by the finding of a large number of false negative GAT test results in acute inflammatory conditions. In such cases, plasma APP concentrations and hematologic findings can provide additional diagnostic information (Table 5). A major disadvantage of the diagnostic use of plasma haptoglobin and SAA is, however, that these analytes currently require use of comprehensive laboratory equipment, which markedly limits their applicability in ambulatory field practice. Notably, the identified cut-off value of ≤14 min was characterized by a specificity of 100% for the prediction of inflammatory disease condition based on the used criteria. This is of interest because previous authors considered GAT coagulation ≤6 min (6) and ≤8 min (4) as clear indicator of inflammation. Moreover, some authors have classified GAT coagulation times ≥10 min as normal (13, 48). Our results suggest that even slightly shortened GAT coagulation times should be already considered as an indicator for the presence of inflammation.

Results from a previous study based on 81 hospitalized cows indicated that haptoglobin and SAA are reliable biomarkers that allowed to discriminate between acute and chronic inflammatory conditions (19). A high diagnostic accuracy was particularly reported for SAA (area under the receiver operating characteristic curve >0.95). These findings were not confirmed in the present study given the observed overlap for SAA and haptoglobin concentrations between cows with acute and chronic inflammatory conditions (Table 5). These diverging results between this former (19) and our study is likely related to differences in size and composition of the study populations, but also to differences in the case definitions used in terms of the chronicity of an inflammatory process. Nevertheless, statistically significantly higher plasma concentrations of SAA were still seen in cows of the present study with acute inflammatory conditions when compared to those with chronic conditions. Furthermore, our data indicate that haptoglobin and SAA concentrations do not change uniformly in case of acute inflammatory disease, as indicated by a lower coefficient of correlation between those variables in cows with an acute inflammatory condition when compared to those with a chronic one. For those reasons, plasma SAA concentrations may have greater clinical utility in cattle with acute inflammatory conditions. A more rapid increase and earlier peak of SAA compared with haptoglobin was also reported in experimental studies in cows after endotoxin challenge (49, 50) and in calves following infection with *Mannheimia haemolytica* (51). This may be attributable to a different responsiveness to pro-inflammatory cytokines and different roles of these two APP in the systemic reaction to inflammation. Serum amyloid A is involved in the clearance of endotoxin, it induces chemotaxis of neutrophilic granulocytes and monocytes, opsonizes bacteria and removes cholesterol from the inflammatory site, whereas haptoglobin has anti-inflammatory, anti-oxidative and bacteriostatic effects (15, 16). Haptoglobin is a binding protein for hemoglobin, released from erythrocytes in circulation. By binding free hemoglobin, it induces a bacteriostatic effect by reducing access of bacteria to hemoglobin-derived iron that is essential for bacterial growth (15, 16, 52).

Although cows with chronic inflammatory conditions had lower plasma SAA concentrations than cows with acute inflammatory condition, increased plasma SAA was still present in most of them. This was not an unexpected finding as increased serum concentrations of SAA were also observed in a previous study on chronic cases of cows with mastitis, metritis, traumatic reticuloperitonitis, and pododermatitis, which were associated with the clinical picture of renal amyloidosis in a small subset of animals (53). This rare condition is related to the deposition of fibrillary amyloid protein aggregates (due to sustained high plasma SAA concentrations) in various organs and tissues (in cattle mainly in the kidney) and is characterized by a nephrotic syndrome with polyuria, polydipsia, proteinuria, edemas, weight loss, hypoproteinemia, and diarrhea (53–55).

Measured concentrations of plasma haptoglobin and SAA concentrations in this study were in part much higher than reported in previous field studies on the diagnostic utility of these biomarkers (24, 30, 31, 56–59). This may partially be due to the inclusion of a highly preselected study population of second-opinion cases referred to a university teaching hospital, which may create a bias toward more severely affected cases. In this context, it needs to be considered that a large proportion of animals suffered from severe septic processes, which included 39 cases of peritonitis (19.3%). However, large variations in APP concentrations were also reported in reviews (17, 18, 60) for animals with similar diseases and even for healthy animals, indicating large variability of results based on the assay used (see also section 2.2). A major limitation of this study is therefore the lack of inclusion of a healthy control group, which required the extraction and use of normal values from the literature for haptoglobin and SAA. Furthermore, our analyses did not allow for comparison of findings between cows with inflammatory and non-inflammatory conditions, as the clinical work-up was problem-based and an inflammatory process could not be completely ruled out even if there was no clinical evidence of inflammation. The use of total serum globulin concentrations as a proxy of the immunoglobulin concentration might be considered as another limitation and it is likely that electrophoretic determination of the gamma-globulin fraction might have provided more accurate results, however this was not possible due to practical constraints in the frame of this study. Also, the discrimination between acute and chronic inflammatory disease condition was largely dependent on the quality of the provided history. An obvious strength of our study is, however, the prospective study design, as well as the relatively large study population and the broad range of laboratory variables analyzed in a standardized manner.

## 5 Conclusion

The findings of the study reported here clearly indicate that there is considerable diagnostic agreement between shortened GAT coagulation times and increased plasma concentrations of haptoglobin and SAA, therefore confirming the clinical utility of this simple cow-side test for diagnosing inflammatory disease conditions. However, results of this study also show that acute inflammatory diseases can result in false negative GAT test results. In such cases, acute phase protein concentrations and hematologic findings can consequently provide valuable additional diagnostic information. Our results further suggest that even a slightly shortened GAT coagulation time should be already considered as an indicator for the presence of inflammation, even though the GAT provides a more reliable indication of inflammation in chronic cases.

## Data availability statement

The raw data supporting the conclusions of this article will be made available by the authors, without undue reservation.

## Ethics statement

The animal studies were approved by Cantonal Animal Testing Commission of the Canton of Bern, Switzerland (permit number BE 47/20). The studies were conducted in accordance with the local legislation and institutional requirements. Written informed consent was obtained from the owners for the participation of their animals in this study.

## Author contributions

FT: Conceptualization, Data curation, Formal analysis, Funding acquisition, Investigation, Methodology, Project administration, Supervision, Validation, Visualization, Writing – original draft, Writing – review & editing. MB: Funding acquisition, Investigation, Writing – review & editing. LP: Investigation, Writing – review & editing. RB: Investigation, Writing – review & editing. MM: Conceptualization, Investigation, Methodology, Project administration, Resources, Writing – review & editing, Supervision.
