# Supplementary material for: Association of results of the glutaraldehyde coagulation test with plasma acute phase protein concentrations and hematologic findings in hospitalized cows
Source: Front Vet Sci. 2024 Jun 19;11:1404809. doi: 10.3389/fvets.2024.1404809 (PMC11220118; doi:10.3389/fvets.2024.1404809)
Supplement: Supplementary file 1 [file Table_1.DOCX]

Supplementary Material

**Supplementary Table 1.** Results of univariable power and linear regression analyses for predicting the coagulation time of a whole-blood glutaraldehyde test (GAT) by means of selected biochemical variables in 120 hospitalized cows with a positive GAT test result (coagulation time ≤ 15 minutes).

| **Variable** | **Model** | **Constant (SE)** | **Slope (SE)** | **R^2^** | ***P*-value constant** | ***P*-value slope** |
| --- | --- | --- | --- | --- | --- | --- |
| Plasma globulin | Power | 321998 (246224) | -2.75 (0.19) | 0.63 | 0.19 | < 0.001 |
| Serum globulin | Power | 25480 (20365) | -2.22 (0.21) | 0.48 | 0.21 | < 0.001 |
| Fibrinogen Clauss | Power | 90.6 (24.4) | -1.54 (0.15) | 0.46 | 0.30 | < 0.001 |
| Fibrinogen Heat | Power | 55.8 (14.4) | -1.17 (0.14) | 0.38 | < 0.001 | < 0.001 |
| Plasma total protein | Power | 59880691 (116010808) | -3.64 (0.44) | 0.37 | 0.61 | < 0.001 |
| Serum total protein | Power | 647715 (1287839) | -2.67 (0.46) | 0.22 | 0.62 | < 0.001 |
| Plasma albumin | Linear | -6.96 (2.31) | 0.48 (0.07) | 0.27 | 0.003 | < 0.001 |
| Plasma A/G - ratio | Linear | -3.66 (0.91) | 18.4 (1.38) | 0.60 | < 0.001 | < 0.001 |

Fibrinogen Clauss = plasma fibrinogen concentration determined with the Clauss method, Fibrinogen Heat = plasma fibrinogen concentration determined with the heat precipitation method, A/G - ratio = Albumin to globulin ratio
